# Association Analyses of Autonomic Dysfunction and Sympathetic Skin Response in Motor Subtypes of Parkinson's Disease

**DOI:** 10.3389/fneur.2020.577128

**Published:** 2020-11-03

**Authors:** Jian-Yong Wang, Meng-Yan Wang, Rong-Pei Liu, Yan Li, Wen-Yuan Zhang, Begench Ovlyakulov, Xiong Zhang, Jian-Hong Zhu

**Affiliations:** ^1^Department of Geriatrics & Neurology, The Second Affiliated Hospital and Yuying Children's Hospital, Wenzhou Medical University, Wenzhou, China; ^2^Department of Preventive Medicine, Wenzhou Medical University, Wenzhou, China

**Keywords:** Parkinson's disease, autonomic dysfunction, sympathetic skin response, motor subtype, asymmetry

## Abstract

**Introduction:** Autonomic dysfunction is a common and disabling non-motor symptom of Parkinson's disease (PD). We aimed to understand autonomic dysfunction in PD motor subtypes, the pattern of sympathetic skin response (SSR) to motor asymmetry, and the association of SSR with autonomic and motor dysfunctions.

**Methods:** A total of 101 PD patients of Han Chinese were included. Unified PD rating scale (UPDRS), scales for outcomes in PD-autonomic symptoms (SCOPA-AUT), orthostatic hypotension, and SSR were evaluated.

**Results:** SCOPA-AUT and incidences of orthostatic hypotension and absent SSR were worse in the subtype of postural instability gait disorder (PIGD) than the subtypes of tremor dominant and intermediate. SSR latency and amplitude were asymmetrical corresponding to the accentuation of motor severity. Patients with absent SSR had worse UPDRS and SCOPA-AUT scores. SSR parameters of the severe side in patients with SSR showed no independent association with the scores.

**Conclusion:** Our results support that autonomic dysfunction is more severe in the PIGD than other subtypes and demonstrate an asymmetry of SSR in PD patients. Absent SSR may indicate worse autonomic and motor symptoms, but SSR parameters are not sufficient to evaluate the severity of the dysfunctions.

## Introduction

Parkinson's disease (PD) is a common neurodegenerative disease caused by the progressive loss of dopaminergic neurons in the substantia nigra pars compacta. Patients not only suffer from motor inconveniences and non-motor symptoms such as cognitive impairment, psychosis, and autonomic dysfunction but also impaired quality of life (1). Similar to motor symptoms, courses, and treatment responsiveness (2), heterogeneity is also present in non-motor symptoms of PD patients. For example, patients of the postural instability gait disorder (PIGD) subtype exhibit more severe sleep problems, fatigue, and urinary symptoms compared to the tremor dominant (TD) subtype (3). When patients develop from other subtypes to the PIGD, the rate of dementia is elevated due to a fast cognitive decline in this subtype (4, 5).

Autonomic dysfunction is one of the most common non-motor symptoms in PD and is considered to be heterogeneous as well (6). All regions of the autonomic system are possibly involved, including the cardiovascular, gastrointestinal, urogenital, thermoregulatory, and pupil (1). While it is well-studied that sleep problem, fatigue, urinary symptom, and cognitive decline are discrepant between PD motor subtypes (3, 7), limited is known in regard with the autonomic dysfunction. Meanwhile, motor symptoms of PD often develop and persist asymmetrically (8, 9), so do some non-motor symptoms such as limb pain and fatigue (10, 11). However, it remains unclear whether the asymmetry resides in the autonomic dysfunction in PD.

Sympathetic skin response (SSR) is a simple and non-invasive method used to evaluate skin sympathetic sudomotor function based on conductance changes in response to stimuli (12, 13). This method can detect bilateral upper and lower limbs separately and is considered less affected by antiparkinsonian drugs and mental stress (14, 15). In this study, we performed a comprehensive evaluation of the autonomic symptoms, including a measurement of SSR, in a group of idiopathic PD patients. We aimed to understand differential autonomic dysfunction in PD motor subtypes, the pattern of SSR in response to PD motor asymmetry, and the association of SSR with autonomic and motor dysfunctions.

## Methods

### Patients

A total of 101 PD patients of Han Chinese ethnicity consisting of 49 men and 52 women were recruited in this study. The mean age of the patients was 65.2 ± 8.3 years. All patients were diagnosed by two movement disorder neurologists according to the UK Parkinson's Disease Society Brain Bank Criteria. Excluded participants were those with a family history of PD, secondary and atypical parkinsonism, or confounding factors potentially involving autonomic symptoms (such as diabetes mellitus, peripheral neuropathy, and low level of vitamin B12), on medications potentially influencing autonomic symptoms (such as tricyclic antidepressants, beta blockers, and anticholinergic drugs), and being diagnosed with cognitive impairment which would otherwise compromise our assessment. All included patients signed written informed consents. This study was approved by the ethics committee of the Second Affiliated Hospital and Yuying Children's Hospital, Wenzhou Medical University.

### Clinical Evaluations

Clinical information including age at onset, disease duration, L-dopa equivalent daily dosage (LEDD), scale for outcomes in PD for autonomic symptoms (SCOPA-AUT), and non-motor symptom assessment scale (NMSS) was collected through face-to-face interviews. Unified PD rating scale (UPDRS), Hoehn–Yahr stage, orthostatic hypotension, and SSR were evaluated 12 h after stopping antiparkinsonian medication, a practically defined OFF medication state (16). The severe and less-severe sides of motor symptoms were determined for each patient by calculating a motor asymmetry score (17). Orthostatic hypotension was defined as a drop in systolic blood pressure for at least 20 mmHg and/or diastolic blood pressure for at least 10 mmHg within 3 min when changing from supine to standing (18). SSRs of bilateral upper and lower limbs were performed using a multichannel computerized electromyograph (Dante Keypoint G4, Natus Medical, Denmark) by a trained neurophysiologist. The stimulator was placed over a median nerve at the wrist with a stimulation intensity at 20 mA for 0.2 ms. Surface electrodes were placed on palms of upper limbs and plants of lower limbs with reference electrodes placed on the dorsal region. The filter setting was 0.5–2,000 Hz. For each limb, three recordings were performed with stimulus intervals more than 30 s. The widest amplitude and its corresponding latency were adopted. The response was considered absent when no change larger than 50 μV was observed in any of the three recordings in 2 s following a stimulus (19).

### Motor Subtypes

Each of the patients was classified into subtypes of tremor-dominant (TD), postural instability and gait disturbance (PIGD), or intermediate as described previously (20, 21). In brief, such assignments were determined based on the ratio of mean UPDRS tremor score (item 16, 20, and 21) to mean UPDRS PIGD score (item 13, 14, 15, 29, and 30). Patients with a ratio of ≥1.5, ≤ 1.0, and in between were defined as TD, PIGD, and intermediate, respectively (20).

### Statistical Analysis

Data were analyzed using the statistical package of Predictive Analytics Software 19.0 (PASW, version 19.0) for Windows. Distribution normality was evaluated by the Kolmogorov–Smirnov test. Differences in gender, orthostatic hypotension frequency, and absent SSR frequency between PD subtypes were assessed using the Chi square test. Differences in age, age at onset, UPDRS-Total score, UPDRS-III score, and SCOPA-AUT score were analyzed by one-way analysis of variance. Differences in disease duration, Hoehn–Yahr stage, LEDD, and NMSS score were analyzed using the Kruskal–Wallis test or Mann–Whitney test. Multivariate analysis was performed by multiple linear regression model or binary logistic regression model using the stepwise forward method with subtypes, gender, age, age at onset, duration, LEDD and UPDRS-III score as covariates. Differences in SSR parameters between the severe and less-severe sides were analyzed by the Wilcoxon signed-rank test. Spearman rank correlation was used to test the association between SSR parameters and scores of SCOPA-AUT or UPDRS-III. A two-tailed *P* < 0.05 was considered statistically significant.

## Results

### Demographic and Clinical Characteristics of the PD Patients

The 101 PD patients comprised 51 PIGD, 33 TD, and 17 intermediate subtypes. Gender, age, age at onset, disease duration, and LEDD were comparable (*P* > 0.05) among the three subtype groups (Table 1). In contrast, significant differences were present in UPDRS-Total score (*P* < 0.001), UPDRS-III (*P* = 0.013), Hoehn–Yahr stage (*P* < 0.001), NMSS (*P* < 0.001), SCOPA-AUT (*P* < 0.001), orthostatic hypotension frequency (*P* = 0.011), and absent SSR frequency (*P* = 0.001). Numeric results of all these measurements were higher in the PIGD subtype compared to the other two subtypes. However, for the ones with SSR latency and amplitude detected, SSRs of the severe side in both upper and lower limbs showed no significant difference among the three subtypes (Supplementary Figure 1).

**Table 1:** Demographic and clinical characteristics of PD patients.

	**PIGD**	**TD**	**Intermediate**	***P***	P^*^
Subject, *n* (%)	51 (50.5)	33 (32.7)	17 (16.8)		
Gender, F/M	26/25	19/14	7/10	0.544^a^	0.554^a^
Age, mean ± SD	66.3 ± 8.0	63.1 ± 8.7	66.1 ± 9.2	0.213^b^	0.086^b^
Age at onset, mean ± SD	61.2 ± 8.6	59.0 ± 9.0	62.8 ± 9.2	0.314^b^	0.262^b^
Duration, years (IR)	5.0 (2.0–7.0)	3.0 (1.0–6.0)	3.0 (1.0–5.0)	0.060^c^	0.068^d^
UPDRS-Total, mean ± SD	34.3 ± 16.4	22.5 ± 12.9	21.5 ± 9.0	<0.001^b^	0.001^b^
UPDRS-III, mean ± SD	19.3 ± 10.9	14.4 ± 8.7	12.5 ± 5.5	0.013^b^	0.034^b^
H-Y stage, median (IR)	2.5 (2.0–2.5)	2.0 (1.0–2.0)	2.0 (1.0–2.0)	<0.001^c^	0.002^d^
LEDD, mg (IR)	450.0 (300.0–601.2)	375.6 (275.0–550.0)	450.0 (350.0–601.2)	0.589^c^	0.452^d^
NMSS, median (IR)	52.0 (33.0–65.0)	17.0 (12.0–29.5)	27.0 (17.5–40.5)	<0.001^c^	<0.001^d^
SCOPA-AUT	13.9 ± 6.3	7.5 ± 4.7	9.3 ± 5.6	<0.001^b^	<0.001^b^
OH, *n* (%)	20 (39.2)	3 (9.1)	5 (29.4)	0.011^a^	0.006^a^
Absent SSR, *n* (%)^e^	37 (18.1)	6 (4.5)	9 (13.2)	0.001^a^	<0.001^a^

*H-Y, Hoehn and Yahr; IR, interquartile range; LEDD, L-dopa equivalent daily dosage; NMSS, non-motor symptom assessment scale; OH, orthostatic hypotension; PD, Parkinson's disease; PIGD, postural instability and gait disturbance; SCOPA-AUT, scales for outcomes in PD-autonomic symptoms; SD, standard deviation; SSR, sympathetic skin response; TD, tremor-dominant; UPDRS, unified Parkinson's disease rating scale*.

* *Compared between the PIGT and TD subtypes*.

a *Analyzed by Chi square test*.

b *Analyzed by one-way analysis of variance*.

c *Analyzed by Kruskal-Wallis test*.

d *Analyzed by Mann-Whitney test*.

e *n and % represent the affected limb number and its percentage relative to the total limb number, respectively*.

### Autonomic Dysfunction in the Subtypes of PIGD and TD

We further analyzed the differences in SCOPA-AUT and orthostatic hypotension between the PIGD and TD subtypes using multivariate analysis adjusted with gender, age, age at onset, duration, LEDD, and UPDRS-III. Results showed significant differences in SCOPA-AUT (B = −4.71, *P* < 0.001) and orthostatic hypotension (OR = 6.452, 95% CI = 1.735–23.988, *P* = 0.005; Table 2) in these two subtypes. An analysis of six individual regions of SCOPA-AUT suggested that differences in autonomic dysfunction were present in gastrointestinal (*P* < 0.001), urinary (*P* = 0.002), cardiovascular (*P* = 0.001) and thermoregulatory regions (*P* = 0.026; Table 3).

**Table 2:** Multivariate risk analysis for SCOPA-AUT and orthostatic hypotension.

**SCOPA-AUT^**a**^**	**B**	***P***	**Orthostatic hypotension^**b**^**	**B**	***P***	**OR**	**95% CI**	
							**Lower**	**Upper**
Constant	3.41	0.500	Constant	−2.30	<0.001	0.100		
Subtypes	−4.71	<0.001	Subtypes	1.86	0.005	6.452	1.735	23.988
UPDRS-III	0.15	0.010						
Duration	0.59	0.001						
Age at onset	0.15	0.033						

*Covariates are subtypes, gender, age, age at onset, duration, LEDD, and UPDRS-III. CI, confidence interval; LEDD, L-dopa equivalent daily dosage; OR, odds ratio; PD, Parkinson's disease; PIGD, postural instability gait disorder; SCOPA-AUT, scales for outcomes in PD-autonomic symptoms; TD, tremor dominant; UPDRS, unified Parkinson's disease rating scale*.

a *Analyzed by multiple linear regression*.

b *Analyzed by binary logistic regression. OR value refers to PIGD vs. TD*.

**Table 3:** Analysis of SCOPA-AUT individual regions between the PIGD and TD subtypes.

**Region (items)**	**PIGD (*n* = 51)**	**TD (*n* = 33)**	***P***
Total autonomic score (23)	13.9 ± 6.3	7.5 ± 4.7	<0.001^a^
Gastrointestinal dysfunction (7)	4 (2–6)	2 (0-3.5)	<0.001^b^
Urinary dysfunction (6)	4 (1–7)	1 (0–3)	0.002^b^
Cardiovascular dysfunction (3)	1 (0–2)	0 (0–1)	0.001^b^
Thermoregulatory dysfunction (4)	4 (1–5)	2 (0–4)	0.026^b^
Pupillomotor dysfunction (1)	0 (0–0)	0 (0–0)	0.894^b^
Sexual dysfunction (2)	0 (0–0)	0 (0–0)	0.868^b^

*PIGD, postural instability and gait disturbance; SCOPA-AUT, scales for outcomes in PD-autonomic symptoms; TD, tremor dominant*.

a *Analyzed by one-way analysis of variance*.

b *Analyzed by Mann-Whitney test*.

### SSR Asymmetry in PD Patients

**Figure 1:**
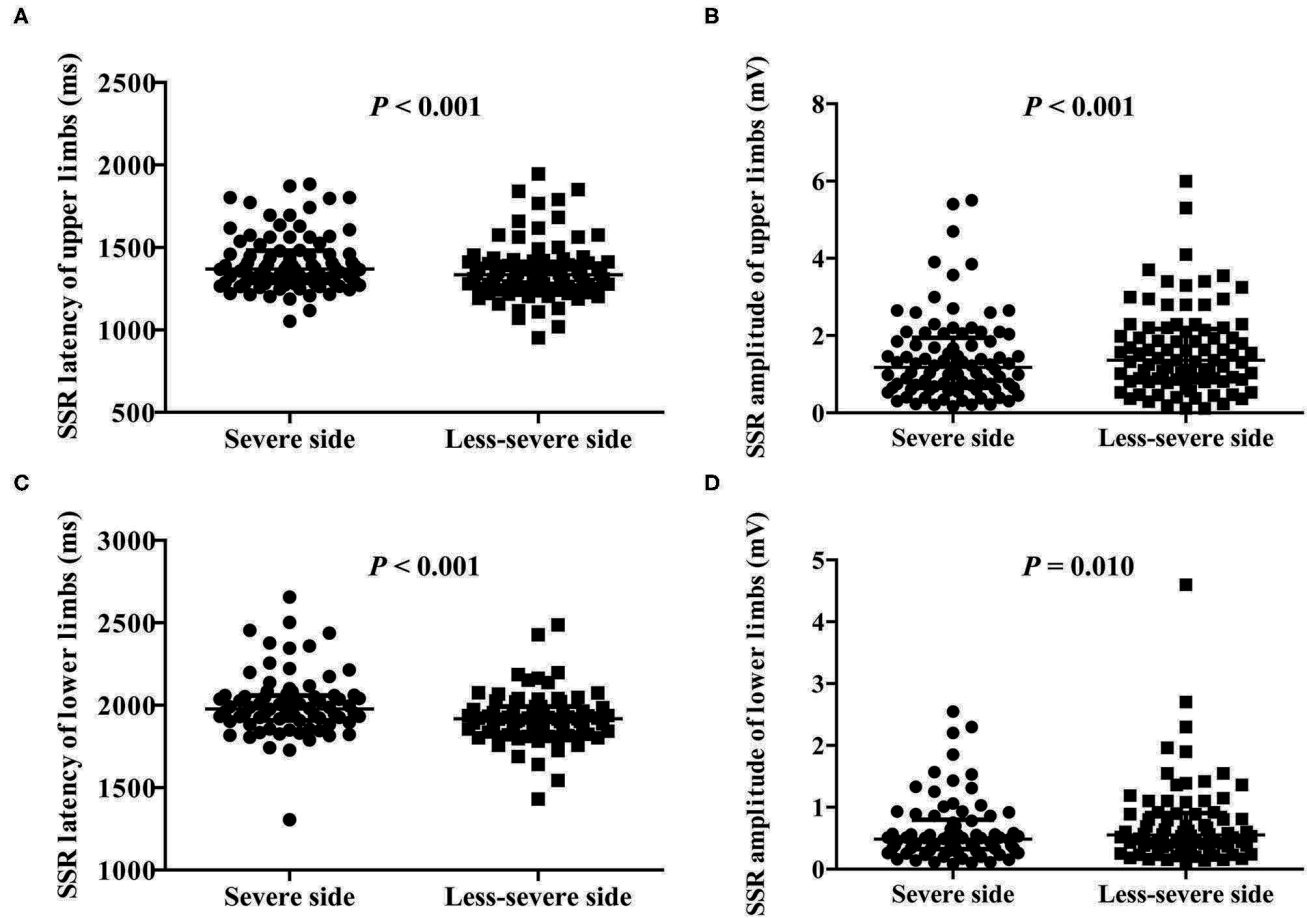
Comparison of SSR parameters between the severe and less-severe sides of motor symptoms. **(A)** SSR latency of upper limbs. **(B)** SSR amplitude of upper limbs. **(C)** SSR latency of lower limbs. **(D)** SSR amplitude of lower limbs. *n* = 89 for upper limbs; *n* = 78 for lower limbs. Values are expressed as median with interquartile range and analyzed with the Wilcoxon signed-rank test. SSR, sympathetic skin response.

In the light of asymmetric motor symptoms in PD patients, we analyzed whether an asymmetry of SSR was present between the severe and less-severe sides (Figure 1). Patients with absent SSR were excluded from the analysis. We showed that latency of the SSR was significantly extended in the severe side of both upper and lower limbs compared to the less-severe side (*P* < 0.001; Figures 1A,C). In line with these observations, amplitude of the SSR was significantly reduced in the severe side of upper and lower limbs (*P* < 0.001 and = 0.01, respectively; Figures 1B,D).

### SSR Correlation Analysis With Autonomic and Motor Symptoms

**Figure 2:**
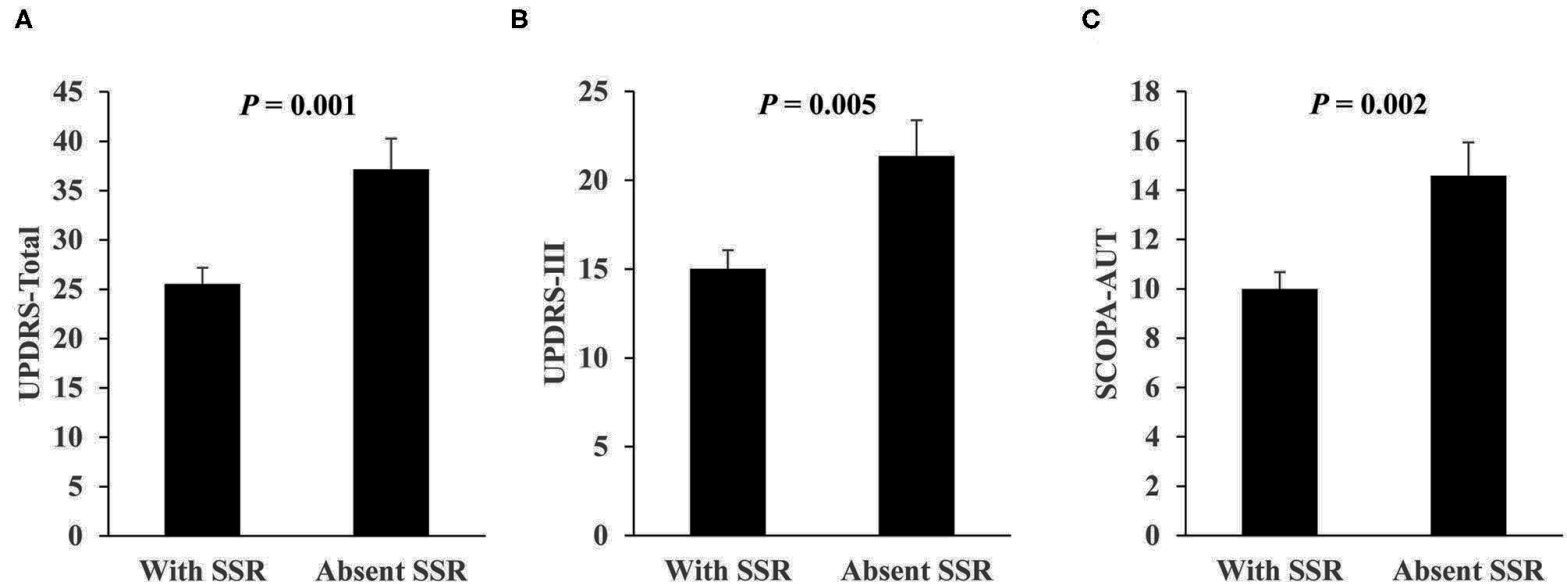
Comparison of UPDRS and SCOPA-AUT scores between PD patients with SSR and with absent SSR. **(A)** UPDRS-Total score. **(B)** UPDRS-III score. **(C)** SCOPA-AUT score. *n* = 77 for with SSR, *n* = 24 for with absent SSR. Values are expressed as mean with standard error and analyzed with one-way analysis of variance. SCOPA-AUT, scales for outcomes in PD-autonomic symptoms; SSR, sympathetic skin response; UPDRS, unified Parkinson's disease rating scale.

The patients with absent SSR had worse UPDRS-Total, UPDRS-III, and SCOPA-AUT scores compared with those with SSR (Figure 2). The latter were included for further correlation analysis of SSR parameters of the severe side with autonomic and motor scores. Results showed that SCOPA-AUT scores were inversely correlated with SSR amplitudes of upper limbs (*r* = −0.218, *P* = 0.040) and lower limbs (*r* = −0.228, *P* = 0.044), but not associated with SSR latencies of both limbs (Supplementary Figure 2). In contrast, no difference was detected in SSR amplitude and latency of both upper and lower limbs between patients with and without orthostatic hypotension (Supplementary Figure 3). Analysis of motor symptoms and SSR parameters suggested that UPDRS-III scores were inversely correlated with SSR amplitudes of upper limbs (*r* = −0.213, *P* = 0.046), but not associated with other ones (Supplementary Figure 2). We also performed correlation analysis of SSR parameters with the age and the disease duration. Results showed that SSR amplitudes and latencies of both upper and lower limbs were associated with age of the patients, but not with the disease duration (Supplementary Figure 4). To clarify whether the above correlations between SSR amplitudes and autonomic or motor symptoms were independent of age, we performed a multivariate linear regression analysis with subtypes, gender, age, age at onset, duration, LEDD, UPDRS-III, and SCOPA-AUT as covariates. Results suggested that neither SCOPA-AUT nor UPDRS-III scores were associated with SSR amplitudes independently. Instead, age remained in association with the SSR parameters (Table 4).

**Table 4:** Multivariate risk analysis for SSR amplitudes of the upper and lower limbs^a^.

**Upper limbs**	**B**	***P***	**Lower limbs**	**B**	***P***
Constant	4.024	<0.001	Constant	1.897	<0.001
Age	−0.040	0.003	Age	−0.020	0.002

*Covariates are subtypes, gender, age, age at onset, duration, LEDD, UPDRS-III and SCOPA-AUT. LEDD, L-dopa equivalent daily dosage; PD, Parkinson's disease; SCOPA-AUT, scales for outcomes in PD-autonomic symptoms; UPDRS, unified Parkinson's disease rating scale*.

a *Analyzed by multiple linear regression*.

## Discussion

Autonomic dysfunction is a frequent and disabling complication of PD. In this study, we analyzed autonomic symptoms in a currently largest group of Han Chinese PD patients. The results disclose a heterogeneity of dysautonomia in PD subtypes, an asymmetry of SSR, and the role of SSR absence and parameters in autonomic and motor symptoms.

PIGD and TD are two typical motor subtypes of PD. A number of studies have demonstrated that certain non-motor symptoms differ among these two subtypes as well as the subtype in between (3, 7, 22–24). Our results of SCOPA-AUT, orthostatic hypotension, and absent SSR reveal such a differential presence, with the worst indices being in the PIGD subtype. The varying disease duration in the subtypes may tilt the balance, whereas the multivariate analysis indicates that the differences of SCOPA-AUT and orthostatic hypotension in the subtypes are independent. A worse presence of SCOPA-AUT in the PIGD is suggested in earlier studies of PD non-dopaminergic manifestations (4, 25). Another study in accordance with our results shows negative correlations between TD/PIGD ratio and frequency of orthostatic hypotension (26). However, it appears that the absent SSR rate has not been sufficiently assessed prior to the current study. For those with SSRs, no difference is found among the three motor subtypes, which is in line with previous results (19, 27). SCOPA-AUT covers gastrointestinal, urinary, cardiovascular, thermoregulatory, pupillomotor, and sexual symptoms and is a reliable questionnaire that evaluates autonomic dysfunction in PD (28). Interestingly, pupillomotor and sexual dysfunctions do not differ between the PIGD and TD subtypes while other symptoms are worse in the former subtype.

While motor manifestations often develop and persist asymmetrically in PD (8, 9), we observe an asymmetry of SSR between the severe and less-severe sides in a total of 89 PD patients after excluding the SSR absent ones. This asymmetry is in correspondence to the accentuation of their motor severity. As a note, SSR is not different between two sides of normal individuals (13). Consistent with our results, results of three previous studies with smaller sample sizes suggest similar asymmetry of SSR. SSR amplitude reduction corresponds to the motor affected side in 25 early-staged idiopathic PD patients characterized by monolateral motor involvement (29). SSRs recorded on the side with accentuated motor symptoms exhibit longer latencies and/or smaller amplitudes in 15 (30) and 50 PD patients (27), respectively. Besides limb SSR, limb pain and fatigue may also be asymmetrical (10, 11). Mechanisms underlying these non-motor asymmetries remain unclear. As a comparison, even the side predominance of motor symptoms is at an early stage to search for clues of genesis. It is proposed that inborn unequal number of dopaminergic neurons and/or different vulnerability to environmental, genetic, and metabolic stress between the bilateral substantia nigra may be involved in the etiology of laterization of motor symptoms in PD (31).

Orthostatic hypotension is a key manifestation of cardiovascular dysautonomia in PD and is believed to be caused by degeneration of postganglionic sympathetic neurons which resulted from alpha-synuclein deposition. Such incidence in PD is around 30–40% (32). However, as reported previously in 46 and 15 patients, respectively (19, 30), we do not find that PD patients with orthostatic hypotension have worse SSR than those without this manifestation. These results indicate that the underlying pathways leading to SSR and orthostatic hypotension may be independent. The SSR of the severe side is used for correlation analysis since it may reflect anomalies with higher sensitivity (30). While PD patients with absent SSR display worse autonomic and motor symptoms compared with those with SSR, we demonstrate that the SSR parameters cannot independently reflect the severity of autonomic and motor symptoms. As a note, age is a rather important factor affecting SSR. Two previous studies with 48 and 58 PD patients, respectively, showed inverse correlations of SSR amplitude with SCOPA-AUT and UPDRS tremor scores (14, 33). On the other hand, Giza et al. (15) studied 29 PD patients without clinical manifestation of dysautonomia and found that their SSR was similar to controls regardless a presence of correlation between UPDRS-III score and SSR. Unfortunately, none of these studies considered the influence of age in their analyses.

In conclusion, our results support that autonomic dysfunction is more severe in patients of the PIGD subtype and demonstrate an asymmetry of SSR in PD. As evidenced by the analyses with SCOPA-AUT and UPDRS-III scores, absent SSR may indicate worse autonomic and motor symptoms, but SSR parameters of the severe side are not sufficient to evaluate the progression of the dysfunctions.

## Data Availability Statement

The original contributions presented in the study are included in the article/Supplementary Materials, further inquiries can be directed to the corresponding author/s.

## Ethics Statement

The studies involving human participants were reviewed and approved by Ethics Committee of the Second Affiliated Hospital and Yuying Children's Hospital, Wenzhou Medical University. The patients/participants provided their written informed consent to participate in this study.

## Author Contributions

J-YW, M-YW, R-PL, YL, and XZ examined the patient and acquired and analyzed all clinical data. J-YW, M-YW, W-YZ, and BO assessed the orthostatic hypotension and sympathetic skin response. J-HZ and J-YW reviewed literatures and drafted the manuscript. XZ and J-HZ supervised the study. All authors read, revised, and approved the final version of the manuscript.

## Conflict of Interest

The authors declare that the research was conducted in the absence of any commercial or financial relationships that could be construed as a potential conflict of interest.
