# Supplementary material for: Association Analyses of Autonomic Dysfunction and Sympathetic Skin Response in Motor Subtypes of Parkinson's Disease
Source: Front Neurol. 2020 Nov 3;11:577128. doi: 10.3389/fneur.2020.577128 (PMC7669620; doi:10.3389/fneur.2020.577128)
Supplement: Supplementary file 1 [file Data_Sheet_1.docx]

**Supplementary Figure 1. SSR parameters of the severe side in three PD subtypes.** (A) SSR latency of upper limbs. (B) SSR amplitude of upper limbs. (C) SSR latency of lower limbs. (D) SSR amplitude of lower limbs. In the order of the PIGD, TD and intermediate subtypes, n = 43, 31 and 15 respectively for upper limbs, n =37, 31 and 11 respectively for lower limbs. Values are expressed as median with interquartile range and analyzed with Kruskal-Wallis test. PD, Parkinson’s disease; PIGD, postural instability and gait disturbance; SSR, sympathetic skin response; TD, tremor dominant.
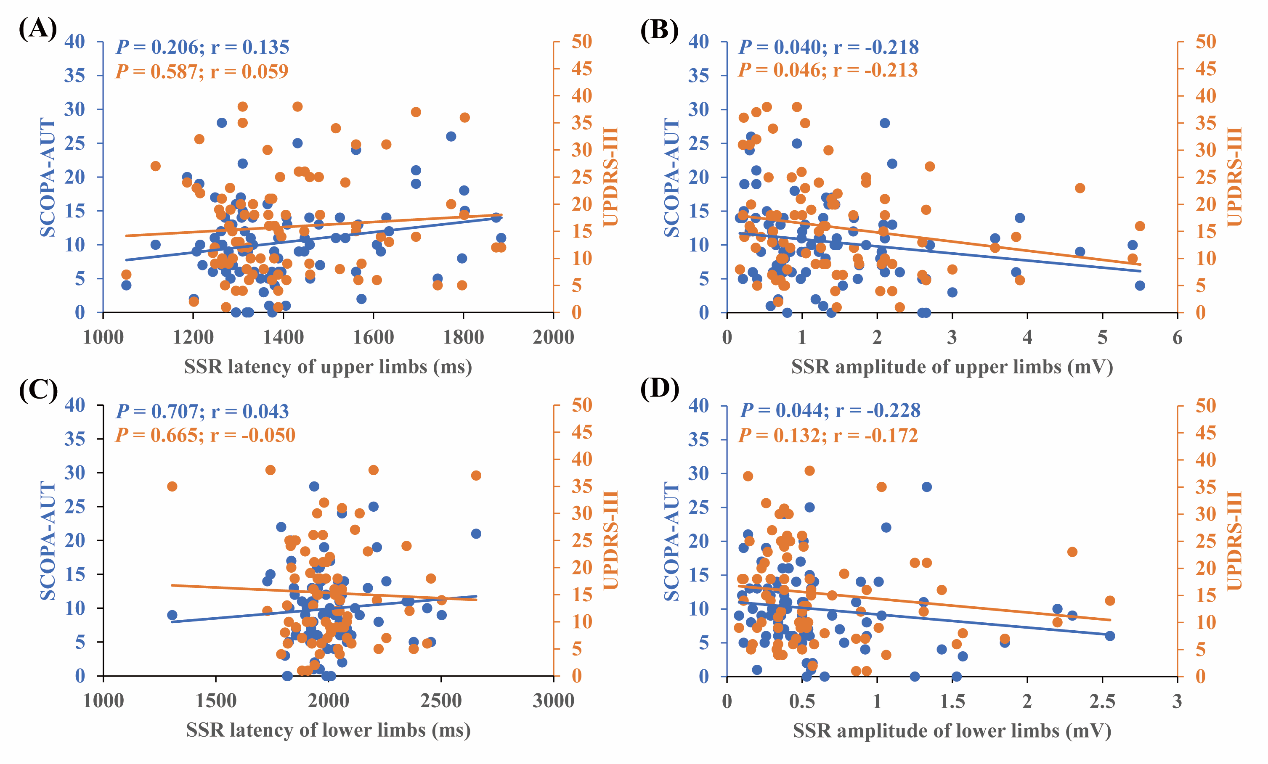


**Supplementary Figure 2. Correlation analysis of SSR parameters of the severe side with SCOPA-AUT and UPDRS-III scores.** (A) SSR latency of upper limbs. (B) SSR amplitude of upper limbs. (C) SSR latency of lower limbs. (D) SSR amplitude of lower limbs. n = 89 for upper limbs, n = 79 for lower limbs. Data were analyzed with Spearman rank correlation test. SCOPA-AUT, scale for outcomes in PD for autonomic symptoms; SSR, sympathetic skin response; UPDRS-Ⅲ, unified Parkinson’s disease rating scale part Ⅲ.





**Supplementary Figure 3. Comparison of SSR parameters of the severe side between PD patients with and without orthostatic hypotension.** (A) SSR latency of upper limbs. (B) SSR amplitude of upper limbs. (C) SSR latency of lower limbs. (D) SSR amplitude of lower limbs. In the order of OH^(+)^ and OH^(-)^, n = 23 and 66 respectively for upper limbs, n = 19 and 60 respectively for lower limbs. Values are expressed as median with interquartile range and analyzed with Mann-Whitney test. OH^(+)^, with orthostatic hypotension; OH^(-)^ , without orthostatic hypotension; PD, Parkinson’s disease; SSR, sympathetic skin response.


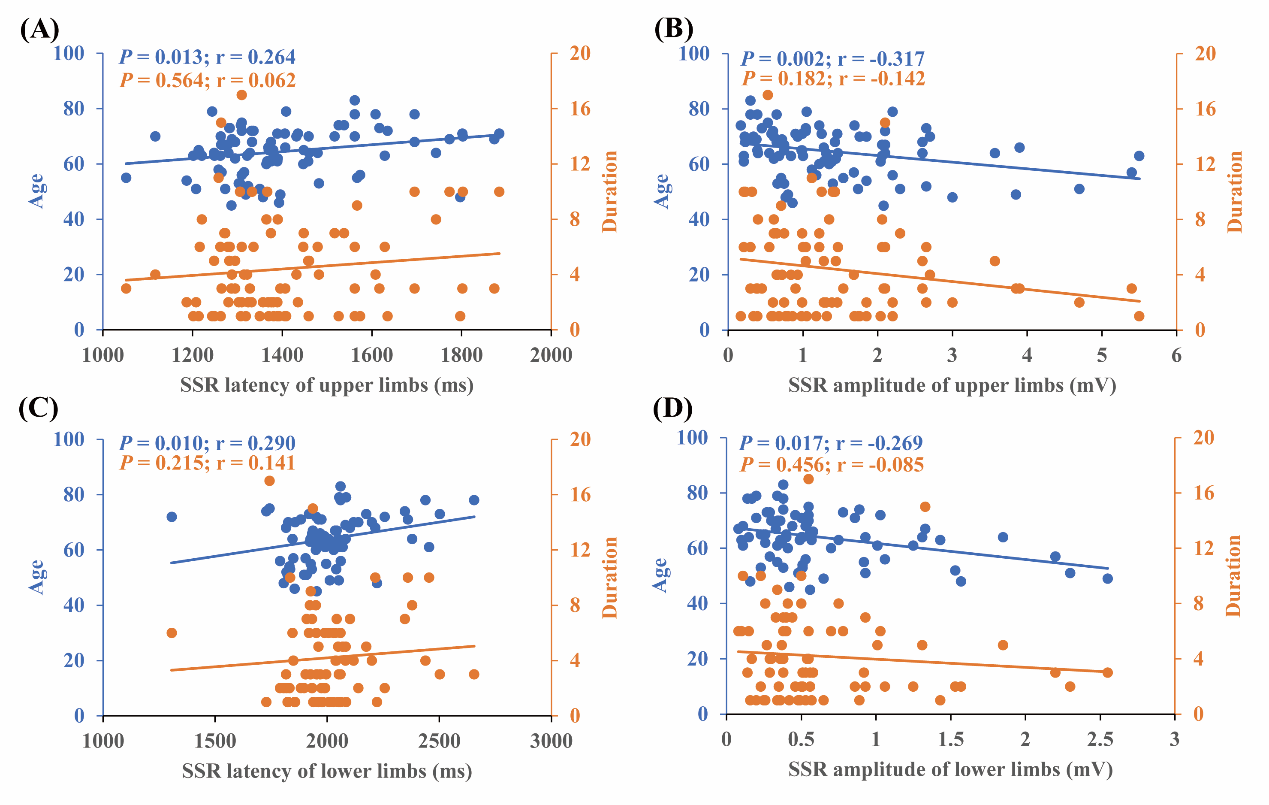


**Supplementary Figure 4. Correlation analysis of SSR parameters of the severe side with age and disease duration.** (A) SSR latency of upper limbs. (B) SSR amplitude of upper limbs. (C) SSR latency of lower limbs. (D) SSR amplitude of lower limbs. n = 89 for upper limbs, n = 79 for lower limbs. Data were analyzed with Spearman rank correlation test. SSR, sympathetic skin response.
